# Maximising Participation in the Australian National Lung Cancer Screening Program: A Discrete Choice Experiment of Eligible, High‐Risk Individuals

**DOI:** 10.1002/resp.70175

**Published:** 2025-12-10

**Authors:** Peiwen Jiang, Caitlin Paton, Richard Norman, Marianne Weber, Henry M. Marshall, Fraser Brims, Kuan Pim Lim, Sarah York, Georgia Bartlett, Richard De Abreu Lourenco, Nicole M. Rankin

**Affiliations:** ^1^ Centre for Health Economics Research and Evaluation University of Technology Sydney Sydney Australia; ^2^ Evaluation and Implementation Science Unit, Melbourne School of Population and Global Health The University of Melbourne Melbourne Australia; ^3^ School of Population Health Curtin University Perth Australia; ^4^ Sydney School of Public Health The University of Sydney Sydney Australia; ^5^ University of Queensland Thoracic Research Centre and Department of Thoracic Medicine The Prince Charles Hospital, Chermside Brisbane Australia; ^6^ Curtin Medical School Curtin University Perth Australia; ^7^ Department of Respiratory Medicine St John of God Subiaco Hospital Perth Australia; ^8^ Albury Private Hospital Clinical Trials Unit Ramsay Health Care Limited Albury Australia

**Keywords:** discrete choice experiment, invitation strategies, lung cancer screening, lung neoplasms, patient navigation, patient preferences

## Abstract

**Background and Objective:**

Relatively little is known about how to maximise participation in lung cancer screening for Australians at high risk of developing the disease. A discrete choice experiment was conducted to elicit and quantify preferences of Australians eligible for lung cancer screening (LCS) to maximise participation in the National Lung Cancer Screening Program (NLCSP) and estimate likely participation.

**Methods:**

Respondents completed an online survey of six LCS factors or ‘attributes’ (invitation to screen, eligibility assessment, appointment booking, model of care, health care worker support and out‐of‐pocket costs). Results were analysed using mixed logit (MIXL), multinomial logit (MNL) and latent class analysis to explore heterogeneity in respondents' choices. Willingness to pay (WTP) for screening attributes were estimated based on the ratio of the coefficient on attributes to cost.

**Results:**

Respondents (*n* = 757) were aged 50–70 years with smoking histories (> 30 pack‐year history and either currently smoke or quit ≤ 10 years). The MIXL showed that participants preferred support from a program navigator, with the highest estimated WTP of $24, plus personalised invitations and lower screening costs. The results identified participation rates that could be achieved through optimal LCS program design, across the most optimistic screening program scenario (87.4%), the scenario proposed in the NLCSP (51.5%) and the least preferred scenario (35.0%).

**Conclusion:**

The results are highly relevant for the NLCSP, which commenced on 1 July 2025. Potential participants place significant value on program navigators, a role not funded within the program, which could significantly improve uptake.

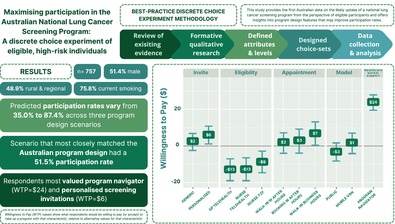

## Graphical abstract

A discrete choice experiment of lung cancer screening attributes. Study results demonstrate that lung cancer screening uptake could be significantly increased by offering program navigators and personalised invitations. The findings contribute significant insights to inform future improvements to the design and implementation of the Australian National Lung Cancer Screening Program.

## 1 Introduction

Lung cancer is the leading cause of cancer‐related death worldwide and accounted for approximately 17% of all cancer deaths in Australia in 2022 [1, 2]. Most lung cancers are attributable to smoking, with 26% of Australians developing the disease by age 80 if they smoked more than 35 cigarettes per day, compared to 1% for people who have never smoked [3]. Lung cancer screening (LCS) using low‐dose computed tomography (LDCT) is an evidence‐based, clinically‐ and cost‐effective intervention in the high‐risk population that significantly reduces lung cancer mortality in randomised controlled trials [4] and real‐world programs [5].

In July 2025, the Australian National Lung Cancer Screening Program (NLCSP) commenced, screening asymptomatic people aged 50–70 years old with a ≥ 30 pack‐year smoking history who either currently smoke or who quit ≤ 10 years prior [6]. Eligible individuals will be offered two‐yearly LDCT screening by a requesting provider (general practitioner [GP], medical specialist or nurse practitioner). Baseline and interval scans will be reimbursed by the government at no cost to the individual [6]. There may be out‐of‐pocket costs for GP appointments; and for those with a high or very high‐risk lung nodule that requires diagnostic investigations, there may be out‐of‐pocket costs for tests, specialist appointments and potentially, treatments such as surgery or radiation therapy. A decentralised approach will enable public and privately owned radiology facilities to offer LDCT scans, with dedicated mobile trucks servicing some rural and remote communities [6, 7].

International evidence shows that LCS program implementation is complex [8]. Breast, bowel and cervical cancer screening programs in Australia use population‐based registers to directly invite eligible people to screen. Wide variation of smoking history data documentation in primary care [9] precludes a standardised approach to identify the eligible population. The NLCSP will use multiple promotion strategies (e.g., media campaigns, information resources and education modules) to raise awareness [6] and address equity. However, the factors that will optimise LCS program participation in Australia are uncertain [10]. A discrete choice experiment (DCE) about LCS conducted in 2020 with 521 Australians at high risk (aged 50–80 years with a history of smoking) identified preferences about the speed of receiving test results (*p* < 0.01), travelling ≤ 50 km, potential LDCT scan costs and radiation exposure [11]. Our team's research on LCS acceptability and feasibility with people who are screen eligible, healthcare providers and policymakers (2022–23) identified complex factors that will likely influence participation in the Australian community [12, 13, 14, 15].

This study aimed to elicit and quantify preferences of people eligible for LCS that will maximise participation and estimate likely participation in the NLCSP via a DCE. A secondary objective was to explore policy‐relevant design features of LCS that might enhance participation in subgroups of respondents.

## 2 Methods

### 2.1 Overview of Approach and Methods

The DCE development drew upon recommended best practice [16, 17] including qualitative research and reviews of existing evidence [8, 18, 19] to guide attribute selection and design. The Research Team was responsible for the survey design approach with input and guidance from a Stakeholder Reference Group.

### 2.2 Rationale for Using Discrete Choice Experiment

A DCE is a quantitative survey method that asks respondents to make choices, under hypothetical conditions, for a health program described by its underlying characteristics, called ‘attributes’ [20]. Respondents are presented with varying combinations of attributes describing different choice options. A significant advantage of DCEs is that, unlike ranking or rating tasks, respondents trade off between competing characteristics. Thus, researchers may gain in‐depth insights into the relative importance of attributes [21]. Results can directly inform policymakers about preferences for incorporation into health programs. Application of DCEs in health are widespread [21, 22] and are valuable in understanding the factors affecting population and preferences for new health programs prior to implementation.

### 2.3 Selection of Attributes

Attribute selection was informed by a four‐stage process framework [23]:

1. *Formative qualitative research to establish a broad range of potential attributes*. There are recommendations for specifying foundational qualitative research for DCEs [16]. An initial set of attributes was derived from: a qualitative interview study of 39 Australian individuals eligible for the International Lung Cancer Screening Trial [12, 24] who had accepted or declined LDCT; and a focus group study conducted with 84 Australian healthcare providers and policymakers [13, 14].
2. *Selection of potential attributes based on modifiable factors*. We grouped factors (*n* = 205) affecting decision‐making under 18 headings [22] using a deductive process where potential attributes were coded as either intrinsic (i.e., not modifiable via policy means) or extrinsic factors for LCS (see Table A1: Supporting Information). The list was reduced by removing intrinsic factors; extrinsic factors (*n* = 161) were reviewed by two authors (C.P., N.M.R.) alongside the literature about LCS barriers and enablers [8, 18]. The Research Team reviewed the candidate list of attributes (*n* = 22). We also reviewed attributes included in two publications about LCS to determine their relevance [11, 25].
3. *Conduct panel meetings for expert review of potential attributes*. We conducted panel meetings with the Research Team and Stakeholder Reference Group to review (a) attribute identification and (b) attribute selection and framing. Seven attributes were selected and further refined for inclusion in the survey. The attribute ‘smoking cessation advice at the initial consultation’ was removed as the expert panel noted that not all respondents would currently smoke. Two consultation sessions were held with consumer representatives.
4. *Refinements to the attribute level descriptors and survey design for pilot testing*. Feedback from panel meetings were finalised by one author (CP) and draft levels for each attribute were prepared based on realistic values extracted from the literature and refined through consensus discussion with the Research Team. Six resulting attributes and levels were pretested in a pilot study (see Table 1).

**TABLE 1:** Final attributes and levels included in the DCE.

Attributes	Levels
Invitation to screen	No invitation^a^
	Generic letter or SMS reminder
	Personalised invitation (letter or SMS)
Appointment booking for the LDCT scan	Walk in availability, 9 AM–5 PM
	Walk in availability, with after‐hours availability
	Book phone/online, 9 AM–5 PM^a^
	Book phone/online, with after‐hours availability
Model of care	Mobile van within local community or township (e.g., car park, school and supermarket)
	Radiology outpatient at community health service or public hospital
	Any radiology site including private clinics or hospitals^a^
Health care worker support	No specific program support beyond your usual provider^a^
	A screening program navigator supporting the entire screening process
Eligibility assessment	Screening program provider via phone/telehealth
	Screening program provider face to face
	GP via phone/telehealth
	GP face to face^a^
Out‐of‐pocket costs for screening	$0^a^
	$50
	$100

*Note*: For all models, usual care was used as the baseline scenario is indicated by for each attribute are: no invitation to attend screening, appointments booking by phone or online during regular business hours, scans conducted at any radiology site including private clinics or hospitals, no specific program support beyond the participant's usual healthcare provider, eligibility assessments conducted in person by a GP and no costs for clients. All attribute levels were dummy‐coded, with the baseline levels assigned a value of ‘0’.

^a^ Indicates the attribute levels for the baseline scenario.

### 2.4 Participant Sampling and Recruitment

We sought to recruit a sample size of approximately 750 participants who would meet the NLCSP eligibility criteria. We sought to oversample participants living in regional, rural and remote areas to ensure broad representation of areas where smoking and lung cancer rates are disproportionately higher than for the general population. DCE sample size calculations are generated based on the number of attributes and levels within each, with a minimum of 20 observations typically needed for each choice set [26]. Participants were invited via a global online consumer research panel, Pureprofile. Pureprofile recruits participants via digital channels such as social media and online advertising. Registered members voluntarily complete surveys in exchange for small incentives, and invitations are sent to those who meet screening criteria and data quality checks.

### 2.5 Survey Design

For DCE items, the experimental design was developed using Ngene software [27] to construct a D‐efficient design, allowing the estimation of main effects. As no prior information on the parameters was available, the design was optimised without incorporating prior assumptions. Twenty unlabelled choice tasks were created and divided into two blocks, with respondents randomly assigned to one of the blocks. Each choice task presented two forced‐choice options, followed by an additional question assessing the respondent's willingness to attend the screening program they selected. Table 2 shows an example choice task.

**TABLE 2:** Walk‐through example of DCE questions.

*Example introductory choice question: ‘At your annual check‐up, your GP has advised you that you are eligible for a new program, a national lung cancer screening program. Your GP outlines the possible options, which option would you choose?’*		
	Option A	Option B
Invite to screen	Generic letter or SMS reminder	Personalised invitation (letter or SMS)
Eligibility assessment	Nurse or other health care provider face to face	Nurse or other health care provider via phone/telehealth
Appointment booking	Walk in availability, 9 AM–5 PM	Book phone/online, with after‐hours availability
Model of care	Any radiology site (this includes private clinics or hospitals)	Mobile van in nearest township (e.g., car park, school and supermarket)
Health care worker support	No specific program support beyond your usual provider/s	A screening program navigator available supporting entire screening process
Cost	$100	$0

Demographic and attitudinal items were included in the survey (see Supporting Information). Items about age and smoking status, including length of time (years), intensity (average number of cigarettes smoked daily) and months/years since quitting filtered out ineligible respondents. Items asked post‐DCE included sex and gender (as per SAGER Guidelines [28]), country of birth and prior medical conditions. Attitudinal items asked a range of topics about primary healthcare use, cancer screening uptake and previous participation in organised programs or opportunistic cancer screening (Table 2).

### 2.6 Statistical Analysis

The choice data were initially analysed using a mixed logit (MIXL) model to account for preference heterogeneity, which allows individual preferences to vary across respondents. Willingness to pay (WTP) estimates were derived from the MIXL model coefficients, providing an assessment of how much respondents are willing to pay for each attribute level. Both continuous and categorical representations of the cost variable were considered in the model to determine the most suitable approach for the final analysis. After comparing the coefficients from both models, the cost variable was treated as categorical. The WTP estimates were then calculated using the average of the cost coefficients [29]. The estimated program participation was derived based on utility values for the most and least preferred programs.

Subsequently, a latent class (LC) model was applied to explore distinct groups of preferences among the respondents. Various LC models with differing numbers of classes were fitted; the optimal number of classes was selected based on the Bayesian Information Criteria. For each respondent, the probabilities of belonging to each class were calculated, and respondents were assigned to the class with the highest probability. Socio‐demographic characteristics were examined across these classes to investigate their relationship with preference heterogeneity. Further analyses, including MNL models and models using cost as a continuous variable were conducted and are provided in Supporting Information. All statistical analyses were performed in R [30] and RStudio [31] using the gmnl package [32]. Demographics and attitudinal items were summarised using descriptive statistics.

Ethical approval was obtained from the University of Melbourne Research Ethics Committee (26732/2023). All respondents completed online consent to participate.

## 3 Results

### 3.1 Pilot Test of Survey

A pilot study involving 76 (10%) respondents was conducted to estimate the preference parameters and timing for completion. No changes were required after pilot testing. The DCE was open for completion online between 15 November 2023 and 4 March 2024. Pureprofile ensured data quality using in‐house quality control tests such as completion time and suspected fraudulent responses.

### 3.2 Sample Characteristics

The survey panel invited 2447 members to participate. Of these, 127 declined consent, 58 did not meet the age‐related criteria and 1505 were excluded based on responses to smoking status items. Consequently, 757 respondents completed the survey. Strict data quality checks were first implemented by the panel company such as speeding, profile validation and bot detection and removal. These were supplemented by internal quality checks, including repeated age verification, a basic mathematics question and a descriptive task involving recognition of a photo of the Australian female soccer team (the Matildas). No respondents failed these internal checks. Median survey completion time was 12 min.

Table 3 presents an overview of participants. The sample was evenly distributed across sex and age groups, and geographical areas (48.9% in regional, rural and remote areas). About three quarters of participants (75.8%) currently smoked, while one quarter (24.2%) had quit smoking ≤ 10 years. Pack‐year history calculations (mean) for these groups were 32.5 and 33.9 years, respectively. The median number of cigarettes smoked per day was 15. Figure B1: Supporting Information shows daily cigarette consumption variation (range 0–60).

**TABLE 3:** Sample demographics.

		Count	Per cent (%)
Age group	50–54 years	227	30.0
	55–59 years	192	25.4
	60–64 years	188	24.8
	65–70 years	150	19.8
Sex	Male	368	48.6
	Female	389	51.4
Indigenous status	Indigenous	29	3.8
	Non‐indigenous	728	96.2
Country of birth	Australia	645	81.4
	Overseas	112	18.6
Education	Completed year 10 or below	188	24.8
	Completed high school or overseas equivalent	142	18.8
	Other training in addition to high school completion	241	31.8
	Undergraduate degree or higher	179	23.6
Geographic region	Metropolitan	387	51.1
	Regional	354	46.8
	Rural or remote	16	2.1
Smoking status	Currently smoke daily	574	75.8
	Have quit smoking within the last 10 years	183	24.2
Disability*	No	547	72.3
	Physical disability	161	21.2
	Intellectual disability	40	5.3
	Prefer not to answer	29	3.8
Health conditions	Chronic Obstructive Pulmonary Disease (COPD)	88	11.6
	Personal history of cancer	90	11.9
	Family history of lung cancer	132	17.4
Healthcare concession card*	No healthcare related concession	326	43.1
	Health care card	184	24.3
	Pensioner concession card (or senior pensioner card)	278	36.7
	Commonwealth seniors' healthcare card	24	3.2
	Veteran card (gold, white, orange)	12	1.6
	Other	5	0.1

* Total per cent equals more than 100 as participants could select more than one response option.

### 3.3 Choice Results and WTP

**FIGURE 1:**
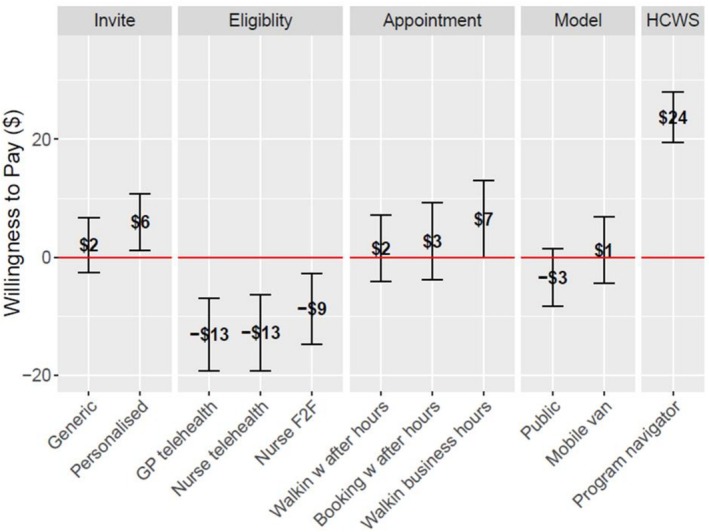
Willingness to pay estimates based on the mixed logit model. WTP estimates with confidence intervals that do not include 0 are statistically significant compared to the baseline.

The MNL and MIXL models (see Table 4) yielded similar results in terms of sign and significance for most parameters and their corresponding WTP values (see Table B1: Supporting Information for the MNL model and Figure B2: Supporting Information). Both models suggested that respondents preferred a program navigator to offer support, personalised invitations, face‐to‐face GP eligibility assessments and reduced screening costs compared to usual care (see Tables B1 and B2: Supporting Information). WTP estimates derived from the MIXL model results are shown in Figure 1. Specifically, respondents were willing to pay $24 for a program navigator to support them through screening and $6 for personalised screening invitations. However, participants needed to be compensated for scenarios where eligibility assessments were conducted via phone or telehealth by GPs, nurses or other healthcare providers relative to usual care. Additional models are included in the Supporting Information.

**TABLE 4:** MIXL results with cost coded as categorical.

	Coef.	SE	*p*
Invite to screen			
Generic letter or SMS reminder	0.039	0.046	0.391
**Personalised invitation (letter or SMS)**	**0.114**	**0.047**	**0.015**
Appointment booking			
**Walk in availability, 9 AM–5 PM**	**0.125**	**0.064**	**0.052**
Walk in availability, with after‐hours availability	0.030	0.055	0.585
Book phone/online, with after‐hours availability	0.053	0.063	0.403
Model of care			
Mobile van within local community or township	0.024	0.055	0.663
Radiology outpatient at community health service or public hospital	−0.065	0.048	0.173
Health care worker support			
**A screening program navigator**	**0.455**	**0.042**	**< 0.001**
Eligibility assessment			
**Nurse or other health care provider via phone/telehealth**	**−0.244**	**0.063**	**< 0.001**
**Nurse or other health care provider face to face**	**−0.167**	**0.059**	**0.004**
**GP via phone/telehealth**	**−0.249**	**0.060**	**< 0.001**
Out‐of‐pocket cost of scan			
**$50**	**−1.913**	**0.163**	**< 0.001**
**$100**	**−1.836**	**0.058**	**< 0.001**

Standard deviation			
Invite to screen			
Generic letter or SMS reminder	0.000	0.089	0.996
Personalised invitation (letter or SMS)	0.190	0.172	0.270
Appointment booking			
Walk in availability, 9 AM–5 PM	0.004	0.089	0.960
Walk in availability, with after‐hours availability	0.211	0.190	0.265
Book phone/online, with after‐hours availability	0.004	0.084	0.963
Model of care			
**Mobile van within local community or township**	**0.572**	**0.068**	**< 0.001**
Radiology outpatient at community health service or public hospital	0.002	0.327	0.995
Health care worker support			
**A screening program navigator**	**0.697**	**0.052**	**< 0.001**
Eligibility assessment			
Nurse or other health care provider via phone/telehealth	0.128	0.261	0.623
Nurse or other health care provider face to face	0.008	0.155	0.958
GP via phone/telehealth	0.005	0.133	0.972
Number of observations			7570
AIC			7695
BIC			7861
LL			−3823

*Note*: Significant attribute levels were bolded.

Abbreviations: AIC, Akaike's information criteria; BIC, Bayesian information criteria; Coef, coefficient estimate; LL, log likelihood; SD, standard deviation.

### 3.4 Preference Heterogeneity

Preference heterogeneity was explored using both LC models and MNLs with respondent characteristics included as interaction terms.

Results from the LC model are presented in Supporting Information with Figure B3 showing:

- Class 1, called the ‘low cost and navigator group’ comprising 68% of respondents who disliked screening programs that involved costs and preferred a program navigator.
- Class 2, called the ‘tailored navigation group’ comprising 32% of respondents who preferred face‐to‐face eligibility assessments, personalised invitations and navigator support, with costs being statistically insignificant. Compared to Class 1, this group was slightly more likely to include females, those aged 50–54, those with higher education and those with prior experience of cancer screening (see Table 5). No significant differences were found between the two classes in terms of country of birth, smoking status, or geographical region.

**TABLE 5:** Estimated uptake rates for three LCS program scenarios.

Attributes	Optimistic	Proposed in NLCSP	Least preferred
Invitation to screen	Personalised invitation (letter or SMS)	No invitation	No invitation
Eligibility assessment	GP face to face	GP face to face	Screening program provider face to face
Appointment booking for the LDCT scan^a^	Walk in availability, with after‐hours availability	Book phone/online, 9 AM–5 PM	Book phone/online, with after‐hours availability
Model of care^a^	Radiology outpatient at community health service or public hospital	Any radiology site including private clinics or hospitals	Mobile van within local community or township (e.g., car park, school and supermarket)
Health care worker support	A screening program navigator supporting the entire screening process	No specific program support beyond your usual provider	No specific program support beyond your usual provider
Out‐of‐pocket costs for screening	$0	$50	$100
Estimated uptake rate	87.4%	51.5%	35.0%

^a^ Attribute levels are not statistically significant.

MNLs were estimated with age, sex and remoteness of residence included as interaction terms to examine heterogeneity arising from these respondent characteristics (Tables D1–D3: Supporting Information). Overall, there were no statistically significant differences in preferences across most attribute levels among demographic subgroups including between females and males. However, some exceptions were observed. Respondents under 60 years of age showed stronger preferences for receiving any type of screening invitation compared with those aged 60 years and older. Regarding remoteness, respondents living in regional areas preferred online or phone booking options with after‐hours availability and were less accepting of additional out‐of‐pocket costs compared with those living in metropolitan areas.

### 3.5 Estimated Program Participation

Table 5 presents estimated uptake rates for three LCS program scenarios (for model estimates, see Table C1: Supporting Information), which were as follows: for the most optimistic scenario, the estimated program participation was 87.4%. For the NLCSP scenario, the rate was 51.5%, while for the least preferred scenario, the estimated participation dropped significantly to 35.0%.

### 3.6 Attitudes to Cancer Screening

Most respondents (89.3%) thought it was very or somewhat important that screening programs detect cancer early. About half preferred an SMS or text message, and a similar proportion preferred a letter as the mode of being reminded for any cancer screening.

Specific items regarding attitudes to LCS included that respondents thought:

- A single invitation at age 50 (55%) and a single reminder once enrolled (59.3%) were the most preferred options, followed by multiple invitations and reminders.
- For additional findings on a LDCT scan, 86.4% indicated it was very/somewhat important to receive information about risk of heart disease, signs of osteoporosis and/or COPD during an appointment where the GP discussed their scan results.
- They would be very likely/likely to participate in an online self‐assessment of LCS eligibility (81.1%), rather than consult a health provider if such an option were available. Online self‐completion was not included in the choice tasks as this is not a feature of the NLCSP.
- Of respondents who reported smoking daily, about 82% were willing to consider best‐practice options for smoking cessation support, while 18% wanted to be able to opt out.

Responses to all attitudinal items can be found in Table A2: Supporting Information.

## 4 Discussion

This study provides the first empirical evidence that quantifies preferences for an Australia NLCSP. The impact of program design on participation was substantial, with predicted participation rates varying from 35.0% to 87.4% across three scenarios. The participation rate for the scenario that most closely matched the NLCSP design was 51.5%. Higher participation rates could be achieved through optimal design that would incorporate multiple implementation strategies including a program navigator, personalised invitations and no out‐of‐pocket costs. Overall, the findings highlight the critical importance of designing programs that meet the preferences of the target population. The sample was highly representative of potentially eligible non‐Indigenous participants in the forthcoming NLCSP in terms of age and smoking history when compared with contemporary modelling estimates of those people who currently smoke (~75%) or have quit smoking (~25%) [10].

A key finding is a strong preference for the LCS program navigator as the most valued program component, with participants willing to pay up to $24 for this support. This figure should be taken at face value and is not a reflection of people's ability to pay for services relative to their income. It reflects heterogeneous willingness to pay across the sample and highlights the potential impact of incorporating navigators into the NLCSP to enhance uptake and facilitate successful implementation.

Evidence from randomised controlled trials (RCTs) demonstrate that navigation models of care are effective in engaging vulnerable populations (including low‐income groups, rural communities, ethnic minorities and individuals facing language barriers) to screen for breast, colorectal and cervical cancer and improving equity [33, 34, 35, 36, 37]. In the LCS setting, a Canadian real‐world pilot program of navigators, 75.5% of participants received a baseline scan in ≤ 12 months and retention was 84.8% for participants requiring a 6‐month follow‐up scan [38]. One RCT found evidence that participant navigation improved LDCT engagement 4.7‐fold among people experiencing homelessness [39], while another demonstrated a significant increase in participation of LDCT scans in low‐socioeconomic communities [36]. This preference in our results may be attributed to the NLCSP not being implemented at the time of the DCE, possibly reflecting a lack of familiarity with program processes and a perceived need for guidance. Further research is warranted to pilot test navigation models of care and report on effectiveness, cost‐effectiveness and implementation outcomes.

Given the variation in preferences observed, the results demonstrate that tailored implementation strategies for different populations are essential to promote program uptake. The results reveal two distinct groups of respondents, the ‘low cost and navigator group’ and the ‘tailored navigation group’. The latter group was slightly more likely to include females, those aged 50–54, those with higher education and those with prior experience of cancer screening. Women in this age group would also be eligible for bowel, breast and cervical cancer screening programs. There is a clear opportunity to test combined screening invitation strategies to enhance participation rates and overall screening experiences. In addition, younger respondents (< 60 years) expressed stronger preferences for receiving screening invitations, whereas older participants appeared less responsive to invitation type. This suggests that invitation‐based outreach may be particularly effective for engaging younger populations, while alternative engagement approaches may be required for older cohorts.

In the NLCSP, the Australian Government will reimburse LDCT scans and fund mobile screening for some rural and remote communities [6]. This is particularly important for populations living in regional areas, who appear more sensitive to costs and less tolerant of additional out‐of‐pocket expenses compared with those living in metropolitan areas. Australia's healthcare system operates as a hybrid model that combines universal health coverage with private insurance, and LDCT scans are most likely to be delivered by private radiology practices. Jurisdictional governments will facilitate LCS delivery in the public sector, predominantly at fixed sites. However, this infrastructure does not guarantee that LCS will be entirely free for participants. GP consultations to obtain an LDCT request form and receive scan results will incur out‐of‐pocket costs if GPs operate mixed‐billing business models; these costs average $45 per consultation in 2023–4 [40]. About 30% of individuals were willing to pay higher costs for more personalised services but many eligible participants may not be willing to, given concerns about the high cost of living and that people who live in outer regional and remote Australia were more likely to smoke daily compared to those living in major cities [41].

When asked about eligibility assessment, participants preferred both GP face‐to‐face consultations and online self‐assessment tools. The preference for GP face‐to‐face consultations was part of the DCE choice tasks and was compared with other options. In the attitudinal items administered post‐DCE, 81% reported being likely to participate in an online self‐assessment of eligibility. This was intentionally omitted as an attribute in the choice tasks because participants recruited from the Pureprofile panel may be more inclined toward online solutions, potentially biasing the DCE results.

The preference for GP consultations in the DCE tasks may reflect trust in the expertise of GPs. About 90% reported utilising primary care; this finding may reflect a belief that face‐to‐face interactions provide a more thorough assessment. In contrast, high acceptance of online self‐assessment indicates a willingness to engage in accessible and time‐efficient options that reduce out‐of‐pocket costs. The findings highlight that flexible pathways for eligibility assessment could be offered; online self‐assessment tools may accommodate diverse preferences and potentially increase NLCSP uptake.

An unexpected finding was that mobile vans were not strongly preferred over usual care (e.g., fixed‐site screening at private radiology services and public hospitals), even among regional and rural respondents. Several factors may explain this finding. First, the lack of geographic consideration in decision‐making may have influenced our results. When making choices, respondents were not explicitly prompted to consider their distance to screening sites, as the relationship between location and screening preferences was not the primary focus of this study. Another Australian DCE on LCS that included distance from home to the screening site found that participants need to be compensated for screening locations 30–50 km away from home [11]. In our study regional and rural respondents may have evaluated mobile screening vans as a general service option rather than considering their practical utility in overcoming geographic barriers.

Second, the DCE design allowed for the co‐occurrence of mobile services alongside high costs in the choice sets, unlike real‐world implementation where mobile screening services for breast and LCS are offered free of charge, which may also have influenced the views about mobile screening in rural and regional respondents.

The findings should not be interpreted as evidence against the use of LCS mobile vans in regional or rural areas. Rather, it highlights a crucial distinction: while mobile vans may be the most practical and accessible option for some communities, they may not be inherently preferred when other options are theoretically available. This insight underscores the need for continued efforts to improve equitable access to screening services in rural and remote areas, whether through mobile services or alternative delivery models.

This study has some limitations. While the sample appears to be broadly representative of eligible participants in terms of age and smoking history, it did not specifically target the priority populations of Aboriginal and Torres Strait Islanders, culturally and linguistically diverse communities or low socio‐economic communities. Given the online methods for recruiting participants and completing the choice sets, the sample may have been more health literate and proficient in English. Future research should explore tailored strategies to address equity concerns to maximise participation of priority populations in the NLCSP.

The study recruited participants exclusively online, potentially biasing the sample toward technology‐literate respondents with higher health literacy, which may have inflated preferences for online features compared to those expected in the general population. As with all stated preference studies, there may be a discrepancy between stated and revealed preferences. Participants may have reported a higher willingness to participate in LCS due to social desirability bias, particularly when costs and potential risks were hypothetical rather than immediate and real.

A limited set of program attributes was included in the study; we may have missed potential factors that could influence screening preferences. However, our study overcame significant limitations previously identified with DCEs, with our team's qualitative research directly informing the selection of potential attributes. The results of these studies show that acceptability depends heavily on program design and implementation strategies [12, 13, 14] while opportunity and capability will directly impact an individual's ability to participate in LCS [12]. We did not replicate attributes already included in the previous DCE [11]. Healthcare systems and prevention strategies (e.g., smoking cessation support) are evolving, meaning our estimated preferences and participation rates represent a snapshot of current preferences. Finally, the results are specific to Australia, and the selected attributes and outcomes may not be generalisable to the healthcare systems and population characteristics of other countries.

In conclusion, the findings of this DCE have important implications for the implementation of the Australia NLCSP and future improvements. High levels of participation could be achieved through optimal LCS program design. Program navigators are an evidence‐based strategy for which respondents expressed the greatest willingness to pay over any other strategy. Navigator models of care require pilot testing and research that demonstrates effectiveness in real‐world settings. Flexible program designs that accommodate different preferences may result in more equitable engagement in screening and require translation into LCS programs internationally.

## Author Contributions

**Peiwen Jiang:** writing – original draft, methodology, software, data curation, validation, formal analysis, visualization. **Fraser Brims:** conceptualization, investigation, funding acquisition, writing – review and editing, methodology. **Nicole M. Rankin:** conceptualization, investigation, funding acquisition, methodology, supervision, resources, project administration, writing – review and editing. **Richard De Abreu Lourenco:** conceptualization, investigation, funding acquisition, writing – review and editing, methodology, formal analysis, software, data curation, supervision, visualization. **Caitlin Paton:** methodology, software, data curation, investigation, validation, formal analysis, writing – original draft. **Kuan Pim Lim:** conceptualization, investigation, funding acquisition, writing – review and editing, methodology. **Henry M. Marshall:** conceptualization, investigation, funding acquisition, methodology, writing – review and editing. **Marianne Weber:** conceptualization, investigation, funding acquisition, writing – review and editing. **Richard Norman:** conceptualization, methodology, investigation, funding acquisition, writing – review and editing. **Georgia Bartlett:** project administration, resources, writing – review and editing. **Sarah York:** methodology, investigation, validation, project administration, resources, writing – review and editing.

## Funding

This work was supported by the (Australian) National Health and Medical Research Council (Ideas Grant Number APP1185390; CI‐A: Nicole M. Rankin). Henry M. Marshall was financially supported by a Metro North Hospital and Health Service (Queensland Government, Australia) Clinical Academic Fellowship and an NHMRC Investigator Grant (Grant Number 1178331). The funders had no role in study design, data collection and analysis, decision to publish or preparation of the manuscript. This publication is solely the responsibility of the contributing authors and does not reflect the views of the funding bodies.

## Ethics Statement

This study was performed in accordance with the Declaration of Helsinki. This human study was approved by the University of Melbourne—approval: 26732/2023. All adult participants provided online informed consent to participate in this study.

## Conflicts of Interest

The authors declare no conflicts of interest.

## Supporting information


**Data S1:** resp70175‐sup‐0001‐Supinfo.docx.

## Data Availability

Individual participant data will not be shared publicly because of privacy or ethical restrictions. Public availability may compromise participants' confidentiality or reveal confidential information.
