# Supplementary material for: Maximising Participation in the Australian National Lung Cancer Screening Program: A Discrete Choice Experiment of Eligible, High‐Risk Individuals
Source: Respirology. 2025 Dec 10;31(4):398–408. doi: 10.1002/resp.70175 (PMC13050626; doi:10.1002/resp.70175)
Supplement: Supplementary file 1 — Data S1: resp70175‐sup‐0001‐Supinfo.docx. [file RESP-31-398-s001.docx]

**Supplementary file**

**Appendix A:** Survey Instrument

## **Page One**

## **Project: A discrete choice experiment on lung cancer screening for high-risk smoking Australians**

**Background:** In May 2023, the Australian Government announced a National Lung Cancer Screening Program, which will commence screening by July 2025. Screening for lung cancer will be targeted at people aged 50 – 70 years who currently smoke (with a smoking history of at least one pack per day for 30 years) or have quit smoking in the past 10 years (having smoked one pack per day for 30 years).

This survey is part of broader research being led by the University of Melbourne and aims to provide evidence to support the roll out of the national lung cancer screening program. In this survey we will present scenarios with different features. For each scenario we would like you to consider if you would participate in the screening program based on the exact information and scenario presented.

If you decide to take part in this study, we will ask you to complete a survey. We anticipate that it will take approximately 15-25 minutes to complete the survey. The survey will ask questions about you and your background (e.g., where you live, your age etc.). This is to make sure that we have participants that are representative of the Australian population.

In this survey we will present you with 10 choice sets. For each choice set we would like you to consider which of the screening options described you would choose to participate in (you can also indicate that you would not participate in screening if neither option were suitable). In each of the choice sets you see; the screening programs are described based on the Invite to screen, Eligibility Assessment, Appointment Booking, Model of care, Health care worker support and Cost of scan. You will be asked to consider what choice you would make in real life.

## **Page Two**

**Part 1: Consent block**

This study is seeking participants who are considered at higher risk for lung cancer. It is possible that reflecting on this risk may cause distress for some individuals. If you feel distressed at any stage reading these materials, please stop the survey and talk with someone.

This can be

- your GP or other health care provider

- You can call BeyondBlue on 1300 224 636 — 24 hours a day, 7 days a week.

- A friend or family member

Do not continue this survey if it increases your distress. Participation is voluntary and you can withdraw at any time.

Please download the following documents so that you can read them later before moving on

Download Consent Information (REQUIRED)

Download Plain Language Statement (REQUIRED)

## **Page Three**

**Part 1: Consent block**

I consent to participate in this survey

1. I understand that this survey is exploring participation in a hypothetical lung cancer screening program.
2. I understand that my participation in this project is for research purposes only and that no screening will be provided to me as a result of my participation in this survey.
3. I understand that if I consent, I will proceed to be asked to complete a number of choice scenarios and be asked to complete a survey with nine parts, that will take approximately 15-25 minutes.
4. I understand that my participation is voluntary and that I am free to withdraw from this survey anytime by contacting the researcher caitlin.paton@unimelb.edu.au.
5. I understand that the survey results will be provided to researchers from the University of Melbourne.
6. I understand that I will not be asked to provide identifiable information including my name or date of birth.
7. I understand that I will be asked questions about my age, sex, gender, and background to ensure that this research includes participants that are representative of the diversity of the Australian population. Data including my smoking history, height, weight, history of cancer, or family history of lung cancer will be used to consider my potential risk of lung cancer for research purposes only. Some demographic data may also be used in the analysis to compare the responses from people in different groups, or with different health needs. This data will not be linked to my personal details, or be identifiable to researchers.
8. I understand that the data from this research will be stored at the University of Melbourne and accessible to University of Melbourne researchers only and will be destroyed 5 years after publication.
9. I have been informed that the confidentiality of the information I provide will be safeguarded subject to any legal requirements; my data will be password protected and accessible only by the named researchers.

- *I consent*
- *I do not consent*

## **Page Four**

**Part 2: Filtering Questions**

The following questions ask you about your age and smoking status to confirm your eligibility for this survey

Please state your age in years

Please describe your smoking status

- I currently smoke daily (includes cigarettes, cigars, pipes, shisha, waterpipe)
- I have quit smoking within the last 10 years
- I have quit smoking more than 10 years ago
- I have never smoked

*Note: Using skip logic, if the participant is not a smoker, or quit more than 10 years ago they will not enter the rest of the survey, they will receive a “Thank you for your time” exit message.*

Please enter the number of years in total that you have smoked

Please enter the average number of cigarettes (or other forms of tobacco such as cigars, shisha etc.) that you smoke each day, or previously smoked each day

Please enter the number of years/months since you have quit smoking

Pilot Explainer

You are completing this survey in the pilot phase. As a result, there are additional questions and space where you can tell us what you are thinking as you answer the survey.

## **Page Five**

**Part 3: Introduction to choices**

In the questions that appear in the next section you will be presented with a series of choices. These choices are only for the purposes of this survey and may be different to the future screening program.

At each question you will need to make a choice between two programs with different features. You can also choose not to participate in either screening program. We would like you to think about each option as if you were actually making the decision about whether or not to participate in this screening program.

There are no right or wrong answers in the choices you make; in each choice set please choose the option that most appeals to you.

## **Page Six**

| **Australian National Lung Screening Program**  The Australian National Screening Program provides screening for people aged 50 to 70 years old who currently smoke (or have quit smoking in the last 10 years). Studies suggest screening with a low dose scan of the chest can reduce lung cancer deaths by at least twenty per cent.  **What does a CT scan involve?**  No preparation is needed. You do not need to fast or get an injection. During the CT scan you will be asked to hold your arms above your head, as well as to hold your breath for a few seconds. The scan generally takes less than a minute to complete, although please allow 30 minutes for your consultation time. If you have had prior chest CT scans notify your doctor and get copies because the radiologists can use these to tell if any findings are new or stable.  **Risks of screening**   - False positive findings may show an abnormal result that turns out to be non-cancerous but may need further diagnostic tests or referral to a specialist. - False negative findings: screening for lung cancer does not detect all lung cancers. - Incidental findings: screening sometimes shows findings in other organs such as the heart. - Radiation risk: the radiation dose of a LDCT scan (1.5mSv) is equal to about 6 months of naturally occurring background radiation, or about 15 x-ray images of the chest - An anxiety resulting from being screened or evaluated for positive or incidental findings may cause significant stress for some patients.   **Results**   - A specialised radiologist will read the CT scan, interpret your scan and send your doctor a report. Your doctor will notify you of the results, discuss the findings with you and organise follow up if needed.   **Smoking**  Smoking is the leading cause of preventable death and disease in Australia. Quitting smoking is associated with a reduced risk of lung cancer along with better outcomes for many health conditions and diseases. Your body starts repairing itself as soon as you’ve smoked your last cigarette.  Calling the Quitline for help can increase your chance of quitting successfully. You can call the hotline on 13 QUIT (13 7848), to talk to a counsellor or request a call back. You can also follow [this link to the QUIT website.](https://www.quit.org.au/articles/about-quitline-13-7848/) |
| --- |

On the next page, you will see an example.

This is the pilot phase of our survey. Please let us know if you have any questions, or suggestions to improve the survey

**Walk- Through Example.** *E.g., A*

| **Program features** | **Option A** | **Option B** |
| --- | --- | --- |
| Invite to screen | Generic letter or SMS reminder | Personalised invitation (letter or SMS) |
| Eligibility Assessment | Nurse or other health care provider face to face | Nurse or other health care provider via phone/telehealth |
| Appointment Booking | Walk in availability, 9am - 5pm | Book phone/online, with after-hours availability |
| Model of care | Any radiology site (this includes private clinics or hospitals) | Mobile van in nearest township (e.g., car park, school, supermarket) |
| Health care worker support | No specific program support beyond your usual provider/s | A screening program navigator available supporting entire screening process |
| Cost of scan | $100 | $0 |

*Which option would you choose?*

- Option A
- Option B
- No screening

**Program Features Explainer –** Note: these should pop up on each attribute as an explanation when someone clicks on it

**Invite to screen** – You may receive an invite to the screening program. This invite can be personalised – which means addressed to you by name, or it could be a generic reminder e.g., a letter that all Australians are sent at age 50.

**Eligibility Assessment** – For the program, a health care provider is required to assess your eligibility based on your age and smoking history. This involves questions around how long you smoked, whether you quit and how long ago you quit, and how much you smoked on average per day.

**Appointment Booking –** This describes how you would make the booking for your lung scan at a radiology service that has a CT scanner. These options explore preferences for walk in bookings, the option to book online, as well as the hours of availability.

**Model of care** – The describes the service available for the lung scan. A mobile van would be available in the centre of regional cities, or rural towns. The other options include attending a public radiology service, such as an outpatient's service at a hospital. The option to attend any radiology site would include all radiology sites with a CT scan, including all private clinics and services.

**Health care worker support** – This describes a navigator who would be a health care professional, who would be available at all stages of screening to provide advice, as a single source of contact for your lung screening journey. This may be by phone, or it may be a role held by a provider you would interact with while attending a mobile van.

**Out of Pocket Cost of Scan** – This describes the total out of pocket cost for up to two appointments with your usual general practitioner, and the lung scan. Up to two GP appointments may be required. One GP appointment would be for a referral for the scan, and another following scan to receive your results.

## **Page Seven+ Eight**

**(choice scenarios will be inserted here)**

Pilot Questions

Choice set 1 –

Do these attributes make sense?

Does the wording of these options make sense?

Choice set 2-10

Do you have any comments on these options?

Does the wording of these options make sense?

At the end of the choice sets

Did these options to choose from make sense?

Would you change any of the program features we are asking you to choose from?

## **Page Nine**

**Part 5: Post choice set questions**

How difficult did you find answering the last XX choice questions that you just answered? Please select one answer

- Very difficult
- Quite difficult
- Nether difficult or easy
- Fairly easy
- Very easy

To what extent did you understand the questions in the previous section? Please select one answer

- I felt that I fully understood all the questions
- I partially understood the questions.
- I did not understand most questions or any questions at all

What did you think about when answering these questions?

## **Page Ten**

**Part 6: Demographic Questions**

The following questions are to consider how your answers would be relevant to other Australian people.

Please state your age in years

Do you identify as any of the following (tick all that apply)?

- Aboriginal
- Torres Strait Islander
- Indian
- Chinese
- Pacific Islander / Māori
- Australian
- Middle Eastern (For e.g., Lebanon, Turkey, Syria or Arabic-speaking)
- British European (For e.g., United Kingdom, Irish)
- European: North-West (For e.g., German, French, Dutch, Austrian, Belgian)
- European: Southern and Eastern (For e.g., Italian, Greek, Croatian, Macedonian)
- South-East Asian (For e.g., Filipino, Indonesian, Vietnamese, Thai)
- North-East Asian (For e.g., Korean, Japanese, Taiwanese – *other than Chinese*)
- Southern and Central Asian (For e.g., Nepalese, Pakistani, Sri Lankan – *other than Indian*)
- People of the Americas (American, Canadian, Latin American, Caribbean, Mexican, all South American ethnic groups)
- North African or Sub-Saharan African
- Other population groups not included elsewhere – free text option.

Do you have a disability? Tick all that apply.

- Physical disability
- Intellectual disability
- No disability
- Prefer not to answer

Do you have known Chronic Obstructive Lung Disease (COPD)?

- Yes
- No
- Prefer not to say

Do you have a personal history of cancer?

- Yes
- No
- Prefer not to say

Do you have a family history of lung cancer?

- Yes
- No
- Prefer not to say

Please enter your weight? *Note: Drop down options, metric*

- Prefer not to say

Please enter your height? *Note: Drop down options, metric*

- Prefer not to say

What is your country of birth?

*Note: Drop down box with all countries, Australia at top*

## **Page Eleven**

What is the highest level of education you have completed?

- Completed Year 9 or below
- Completed Year 10 or overseas equivalent
- Completed high school or overseas equivalent
- Other training in addition to high school completion
- Undergraduate degree or higher
- Prefer not to answer

Do you have a health care card, senior pensioners health care card or other concession card?

- Health care card
- Senior pensioners health care card
- Commonwealth seniors’ health card
- Pensioner concession card
- Veteran Gold card
- Veteran White card
- Veteran Orange card
- Other
- No health care related concession

Do you have a regular General Practitioner?

- Yes
- No
- I attend a practice without having a regular single person
- I attend two or more clinics for different purposes
- Other
- Prefer not to answer

What is the out-of-pocket cost to attend your preferred General Practitioner?

Enter an exact number or choose an option.

- $0 or bulk-billed
- $1-25
- $26-39
- $40-59
- $60 or more
- I do not attend a General Practitioner or have not done so in the last 2 years
- Prefer not to answer

## **Page Twelve**

There are differences in health outcomes based on both sex and gender. This study will follow best practice for data analysis by *both* sex and gender.

Please provide your sex assigned at birth:

- Male
- Female
- Intersex
- Other
- Prefer not to answer.

Please describe your gender identity (select all that apply):

- Woman
- Man
- Transgender
- Non-binary/non-conforming
- Prefer not to answer.

Which of these options best describes where you live most of the time?

- Inner city or metro areas
- Suburban or Outer urban areas
- Regional
- Rural
- Remote
- Other – please describe.

## **Page Thirteen**

**Part 7: Attitudes to screening**

Do you participate in other cancer screening ? Please tick all that apply, including any you have previously participated in:

- BreastScreen Australia Program (this is where you get a mammogram)
- National Cervical Screening Program (Previously known as a Pap test, now HPV test)
- National Bowel Cancer Screening Program (Home ‘poo’ test kit and for some people, colonoscopy)
- Prostate screening with GP (usually a blood test)
- Skin cancer screening (an appointment where your GP, a doctor at skin cancer clinic or a dermatologist checks your skin for moles and may take photographs)
- Other – please provide details
- No, I do not participate in any other screening programs
- Prefer not to answer.

What reminders would you prefer to receive for a screening program? (tick all that apply)

- Letter
- SMS or text message
- Phone call
- Advised when I attend GP

Would you like to be reminded of your eligibility for lung screening? (tick all that apply)

When aged 50 years old

- Single invitation
- Multiple invitations
- No invitation at all
- Prefer not to answer

Once participating in screening

- Single reminder when due every two years
- Multiple reminders when due every two years until I have screened
- No reminders at all
- Prefer not to answer
- I would not participate in lung screening

If the lung cancer screening program offered online self-assessment (about whether you are eligible) so that you could simply attend a scan and only see your GP for the results afterwards, how likely would you be to participate?

- Not at all
- Somewhat unlikely
- Neutral, Unsure
- Somewhat likely
- Very likely
- Prefer not to answer

For some people, how and when they receive advice to quit smoking could influence their decisions to engage in screening. Which of the following best describes your preferences?

- Option to opt out of smoking cessation support
- Referral to QUITLINE
- Referral to discuss further with your GP or other regular health provider
- Offered face to face counselling and nicotine replacement therapy on the day on the scan
- No preference from above

How important do you think screening programs are to detect cancer early?

- Not at all important
- Not very important
- Neutral /not sure
- Somewhat important
- Very important

In addition to checking the health of your lungs, it is possible for the doctor reporting on the results of your scan to provide your GP with the following

- Your risk of heart disease (prediction for the five years) and advice on medication to take
- If you have any signs of osteoporosis
- If you have signs of COPD

How important is it to you, to know this information?

- Not at all important
- Not very important
- Neutral /not sure
- Somewhat important
- Very important

## **Page Fourteen**

**Part 8: Final Questions**

Please enter the **postcode** of the location you live most of the time

Please enter your **year** of birth:

What is the answer? 8+(2x6) =?


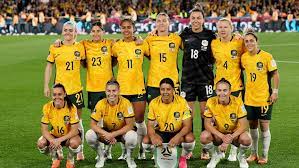


What is this?

**Final Pilot Question**

Thank you for participating in the pilot of our survey. Do you have any further suggestions for changes we should make to the survey?

## **Page Fifteen**

**Part 9: Email for research results or further research**

As screening for lung cancer becomes available in Australia, the researchers would like to follow up this study with further research. Please indicate below if you would like to be contacted about opportunities to be involved in future research. This option is voluntary. If you consent to be contacted in future, please provide your email address when prompted.

Note: This email address will be kept confidential. This will be stored securely consistent with the Data Management Plan for the project. You will not be contacted for any reason other than future research that has been approved by an ethics committee.

- *I consent to be contacted for future research related to lung cancer screening only*
- *I do not consent to any future contact.*

Please indicate if you would like to receive an update of the research results by email when they are complete.

- *I would like an update of the research results by email*
- *I do not want an update.*

## **Page Sixteen**

Email address:

*Note: The survey will only ask for an email address using skip logic, if the participant has consented to further contact or would like an update of the research then they will be asked to provide an email address. Participants will not be able to provide an email address if not required.*

## **Page Seventeen**

The survey is now complete, and your responses have been submitted!

Thank you for taking the time to complete this survey.

Table A1: All intrinsic and extrinsic items elicited from qualitative data for attribute consideration

| **Thematic codes from motivation to screen by Dunlop et al. 2021** | **INTRINSIC / EXTRINSIC** |
| --- | --- |
| Theme: Reasons for Attending |  |
| 1. Worry, concern and blame: Concern about own health, self-blame, fears/not about the test | Intrinsic |
| 1. Helping others: Altruism, contributing to research, family’s future health | Intrinsic |
| 1. Family and family history: Smoking history, relationships, impact of discussions | Intrinsic |
| 1. Practical barriers/facilitators: Proximity to screening, travel, accommodation, cost, incentives to attend, holiday, knowledge, education | Extrinsic |
| Theme: Attitudes |  |
| 1. Belief/disbelief in lung cancer screening | Intrinsic |
| 1. Views on lung cancer and smoking: Fears, beliefs about lung cancer, relationship of lung cancer and smoking, concerns about smoking | Intrinsic |
| 1. Worry about risk: Feelings about own risk, understanding of risk | Intrinsic |
| 1. Personal experience with lung cancer: Family and friends with lung cancer | Intrinsic |
| 1. Impact of health professional: Recommendations, impact of relationship | Extrinsic |
| Responsibility |  |
| 1. Responsible for own health: Who is responsible, strategies to manage, other views, personal control | Intrinsic |
| Reaching the Community |  |
| 1. Challenges: Changes in communication, individual preference, knowledge | Intrinsic |
| 1. Raising awareness: Strategies, personal experience, how did they hear about it | Extrinsic |
| **CAPABILITY** |  |
| 1. Physical Practical Barriers of everyday | Extrinsic |
| 1. Psychological Knowledge and understanding | Intrinsic |
| 1. Self-efficacy | Intrinsic |
| **OPPORTUNITY** |  |
| 1. Physical Location as a barrier | Extrinsic |
| 1. Social: Support from family | Intrinsic |
| 1. Stigma is ever present | Intrinsic |
| 1. Access to a General Practitioner | Extrinsic |
| **MOTIVATION** |  |
| 1. Automatic: Impact of lived experience | Intrinsic |
| 1. Fatalism | Intrinsic |
| 1. Reflective: Awareness of own risk | Intrinsic |
| 1. Screening as beneficial | Intrinsic |
| **THEMATIC CODES from Healthcare provider docus groups: Dodd et al.** |  |
| Enthusiasm for Screening |  |
| 1. Already discussions internally - politics | Intrinsic |
| 1. COVID shown us what we can do from distance | Extrinsic |
| 1. Ex-smokers keen | Intrinsic |
| 1. Invest money elsewhere | Extrinsic |
| 1. Involve target population early in planning | Extrinsic |
| 1. Lung cancer survivors | Intrinsic |
| 1. Potential uptake | Intrinsic |
| 1. Screening fatigue | Intrinsic |
| 1. Smoker motivation | Intrinsic |
| 1. Support from health professionals | Intrinsic |
| 1. Using X-ray to screen | Extrinsic |
| Naming the Program |  |
| 1. Compare to other cancer screening programs | Extrinsic |
| 1. Don't use the terminology ‘cancer’ | Extrinsic |
| 1. Lung Health Check (UK name) | Extrinsic |
| 1. Name should reflect what it is – cancer screening | Extrinsic |
| 1. Need consumer perspective | Extrinsic |
| 1. Overall lung check | Extrinsic |
| 1. Public understanding of screening | Intrinsic |
| Engagement and Awareness |  |
| 1. Advertise in different languages | Extrinsic |
| 1. Advertise to people with high-risk | Extrinsic |
| 1. Co-design | Extrinsic |
| 1. Education needed | Extrinsic |
| 1. Eligibility criteria | Extrinsic |
| 1. External to GP | Extrinsic |
| 1. Funding | Extrinsic |
| 1. GP office, resources | Extrinsic |
| 1. Image of lung cancer | Extrinsic |
| 1. Investment | Extrinsic |
| 1. Mail, social media, sponsored adverts | Extrinsic |
| 1. Normalisation over time | Extrinsic |
| 1. Piggyback on other campaigns | Extrinsic |
| 1. Public transport, QR codes | Extrinsic |
| 1. Targeted adverts on cigarette packaging | Extrinsic |
| 1. Traditional advertising, TV newspapers | Extrinsic |
| Communication and Outreach |  |
| 1. Build trust | Intrinsic |
| 1. Consumer input | Extrinsic |
| 1. Educate everyone | Extrinsic |
| 1. Marketing is key | Extrinsic |
| 1. Need multifaced ways to reach population | Extrinsic |
| 1. Outreach to all, not just those high risk | Extrinsic |
| 1. Participant champions | Extrinsic |
| 1. Simple methods | Extrinsic |
| 1. Understand the population | Extrinsic |
| Recruitment |  |
| 1. Digital methods | Extrinsic |
| 1. Direct letters | Extrinsic |
| 1. Doorknocking rural, regional | Extrinsic |
| 1. Grassroots, population groups | Extrinsic |
| 1. Incentives | Extrinsic |
| 1. Key recruitment messages | Extrinsic |
| 1. No wrong door | Extrinsic |
| 1. Opportunistic | Extrinsic |
| 1. Patient navigators | Extrinsic |
| 1. Primary care | Extrinsic |
| 1. Quitline referral | Extrinsic |
| 1. Risk assessment tool | Extrinsic |
| 1. Self-referral into program | Extrinsic |
| 1. Suggestions | Extrinsic |
| 1. Take screening to the public | Extrinsic |
| 1. Understanding eligibility, simpler risk assessment tool, two stage | Extrinsic |
| Priority Populations |  |
| 1. CALD | Extrinsic |
| 1. Capture motivated | Extrinsic |
| 1. Characteristics | Extrinsic |
| 1. Complex patients | Extrinsic |
| 1. COVID impact | Extrinsic |
| 1. Decide populations | Extrinsic |
| 1. Don't go to GP | Intrinsic |
| 1. Empowerment, alternative pathways | Extrinsic |
| 1. Fear, fatalistic | Intrinsic |
| 1. Full support | Extrinsic |
| 1. Health literacy | Extrinsic |
| 1. Indigenous communities | Extrinsic |
| 1. Indigenous people have mistrust | Intrinsic |
| 1. Local buy-in | Extrinsic |
| 1. Low SES | Extrinsic |
| 1. Multipronged approach to engage | Extrinsic |
| 1. Nihilism | Intrinsic |
| 1. Opportunistic screening | Extrinsic |
| 1. Opportunity for intervention | Extrinsic |
| 1. Participant to be proactive | Intrinsic |
| 1. Psychosocial impact | Intrinsic |
| 1. Relationships with primary care | Intrinsic |
| 1. Rural remote location | Extrinsic |
| Access and Equity |  |
| 1. Access to scanners | Extrinsic |
| 1. Access to treatment | Extrinsic |
| 1. Autonomy (to choose to scan) | Intrinsic |
| 1. Bring program to the people | Extrinsic |
| 1. Convenience of screening | Extrinsic |
| 1. Digital literacy and equity | Intrinsic |
| 1. Ensuring those most at risk can get access | Extrinsic |
| 1. Inner city more awareness of risk | Intrinsic |
| 1. Mobile screening vans | Extrinsic |
| 1. Multipronged approach to outreach | Extrinsic |
| 1. Not exacerbate inequities | Extrinsic |
| 1. Radiology more expensive, harder to access regional | Extrinsic |
| Delivery of the Program |  |
| 1. Access as a key concern | Extrinsic |
| 1. Agreed (national) protocol | Extrinsic |
| 1. American experience (poor uptake) | Extrinsic |
| 1. Bridge government to clinical (expertise) | Extrinsic |
| 1. Federal and state divide | Extrinsic |
| 1. Gradual (phased) implementation | Extrinsic |
| 1. Harness lessons from other screening programs | Extrinsic |
| 1. Key Performance Indicators for screening programs | Extrinsic |
| 1. Lessons from COVID | Extrinsic |
| 1. Limited resources | Extrinsic |
| 1. Link with local clinics and early assessment clinics | Extrinsic |
| 1. Logistics of operation | Extrinsic |
| 1. Need champions | Extrinsic |
| 1. Need infrastructure and data management | Extrinsic |
| 1. Population based screening framework | Extrinsic |
| 1. Recognise access challenges of each state | Extrinsic |
| 1. Referral for incidental findings | Extrinsic |
| 1. Start with those hard to reach | Extrinsic |
| 1. Support for UK model | Extrinsic |
| 1. Telehealth | Extrinsic |
| 1. Well defined pathway | Extrinsic |
| Smoking Cessation |  |
| 1. Comparisons | Extrinsic |
| 1. Consenting to program | Extrinsic |
| 1. Consider stigma | Intrinsic |
| 1. Consumer input | Extrinsic |
| 1. Cost to set up | Extrinsic |
| 1. Downstream impact of referral out of program | Extrinsic |
| 1. Emotional state | Intrinsic |
| 1. Existing programs | Extrinsic |
| 1. Family pressure | Intrinsic |
| 1. GP time restraints | Extrinsic |
| 1. Impact screening | Extrinsic |
| 1. Incentives | Extrinsic |
| 1. Lack of consensus about inclusion (of interventions) | Extrinsic |
| 1. Motivation of screeners | Intrinsic |
| 1. Need support from primary care | Extrinsic |
| 1. Not part of LCS | Extrinsic |
| 1. Opt-in or opt-out models of cessation | Extrinsic |
| 1. Package of care | Extrinsic |
| 1. Perception of gen pop | Extrinsic |
| 1. Reduce smoking when come back for screening | Extrinsic |
| 1. Research evidence | Extrinsic |
| 1. Right thing to do | Intrinsic |
| 1. Screening may undermine smoking cessation | Extrinsic |
| 1. Services for ineligible participants | Extrinsic |
| 1. Smokers resist change | Intrinsic |
| 1. Smokers want to quit | Intrinsic |
| 1. Some will be ex-smokers | Extrinsic |
| 1. Supplying patches | Extrinsic |
| 1. Tailored program | Extrinsic |
| 1. Teachable moment | Extrinsic |
| 1. Timing of introducing smoking cessation | Extrinsic |
| 1. Tobacco control | Extrinsic |
| 1. Training for healthcare providers | Extrinsic |
| Referral across Program |  |
| 1. Central database for scan access | Extrinsic |
| 1. Centralise the system | Extrinsic |
| 1. Entry into lung or imaging MDT | Extrinsic |
| 1. Entry points | Extrinsic |
| 1. GP | Extrinsic |
| 1. Incidental findings | Extrinsic |
| 1. Need appropriate follow up, guidelines | Extrinsic |
| 1. Need timely referral for investigations | Extrinsic |
| 1. Specialist interventions | Extrinsic |
| 1. Supportive follow up structure | Extrinsic |
| 1. Timeline, targets | Extrinsic |
| 1. Who communicate screening results | Extrinsic |
| 1. Work with health structures already in place | Extrinsic |
| 1. Cost-effectiveness |  |
| 1. Are there better ways to reduce LC | Extrinsic |
| 1. Balance initial investment with long term cost savings | Extrinsic |
| 1. Cost to set up program | Extrinsic |
| 1. Costs of outreach | Extrinsic |
| 1. Funding model for centres offering LCS | Extrinsic |
| 1. Medicare for more than just scan | Extrinsic |
| 1. Opportunity costs | Extrinsic |
| 1. Out of pocket costs | Extrinsic |
| 1. Personal and emotional cost | Intrinsic |
| 1. Reduce use of expensive treatment | Extrinsic |
| 1. Self-referral reduces costs | Extrinsic |
| 1. Use of existing resources to save costs | Extrinsic |
| Complexity of Health System |  |
| 1. Competition between screening programs | Extrinsic |
| 1. Federal and state divide | Extrinsic |
| 1. Harder to access radiology in regional areas | Extrinsic |
| 1. Healthcare administration | Extrinsic |
| 1. Involve all stakeholders | Extrinsic |
| 1. LC heterogenous | Extrinsic |
| 1. Public and private providers | Extrinsic |
| 1. Resistance to LCS | Extrinsic |

**Table A2. Items about attitudes towards primary care, cancer screening and lung cancer screening including participation**

| **Category** | **Variable** | **Count** | **Per cent (%)** |
| --- | --- | --- | --- |
| Regular GP* | Yes | 583 | 77.0 |
|  | No | 65 | 8.6 |
|  | Attend a practice but do not have a single GP | 85 | 11.2 |
|  | I attend two or more clinics for different purposes | 9 | 1.2 |
|  | Other | 4 | 0.5 |
|  | Prefer not to answer | 11 | 1.5 |
| Out-of-pocket costs to attend preferred GP*  (n=683) | $0 or bulk-billed | 441 | 64.6 |
|  | $1-$25 | 20 | 2.9 |
|  | $26-39 | 52 | 7.6 |
|  | $40-$59 | 87 | 12.7 |
|  | $60 or more | 52 | 7.6 |
|  | I do not attend a GP | 23 | 3.4 |
|  | Prefer not to answer | 8 | 1.2 |
|  | No response | 74 | 9.8 |
| Participation in other cancer screening programs* | No, I do not participate | 284 | 37.5 |
|  | National Bowel Cancer Screening Program | 322 | 42.5 |
|  | BreastScreen Australia Program | 247 | 63.5^#^ |
|  | Skin cancer screening | 171 | 22.6 |
|  | National Cervical Screening Program | 158 | 40.6^#^ |
|  | Prostate screening with GP | 79 | 21.5^†^ |
|  | Other | 11 | 1.5 |
|  | Prefer not to answer | 6 | 0.8 |
| Perceived importance of screening programs to detect cancer early | Very or somewhat important | 676 | 89.3 |
|  | Neutral /not sure | 73 | 9.6 |
|  | Not very important or not at all important | 8 | 1.1 |
| Mode of reminder for any cancer screening* | SMS or text message | 393 | 51.9 |
|  | Letter | 373 | 49.3 |
|  | SMS or text message or letter | 97 | 12.8% |
|  | Advised when I attend GP | 179 | 23.6 |
|  | Phone call | 43 | 5.7 |
| **LCS specific items** |  |  |  |
| Likelihood of participating in online eligibility assessment for LCS | Very likely or somewhat likely | 614 | 81.1 |
|  | Neutral or Unsure | 90 | 11.9 |
|  | Somewhat unlikely or not at all | 48 | 6.3 |
|  | Prefer not to answer | 5 | 0.7 |

| **Category** | **Variable** | **Count** | **Per cent (%)** |
| --- | --- | --- | --- |
| Preferences for smoking cessation inclusion in LCS^^^ | Referral to discuss further with your GP or other regular health provider | 117 | 18.5 |
|  | Best evidenced support such as face to face counselling and nicotine replacement therapy (NRT) on the day on the scan | 110 | 17.4 |
|  | Referral to QUITLINE with script for NRT | 79 | 12.5 |
|  | No preference from above options | 211 | 33.4 |
|  | None - option to opt out of smoking cessation support | 115 | 18.2 |
| Frequency of reminder at 50 years old for LCS | Single invitation | 416 | 55.0 |
|  | Multiple invitations | 234 | 30.9 |
|  | No invitation at all | 84 | 11.1 |
|  | Prefer not to answer | 23 | 3.0 |
| Frequency of reminder once enrolled in LCS | Single reminder when due every two years | 449 | 59.3 |
|  | Multiple reminders when due every two years until I have screened | 218 | 28.8 |
|  | No reminders at all | 48 | 6.3 |
|  | Prefer not to answer | 42 | 5.5 |
| Importance of GP providing information about risk of heart disease, signs of osteoporosis and signs of COPD | Very or somewhat important | 654 | 86.4 |
|  | Neutral /not sure | 79 | 10.4 |
|  | Not very important or not at all important | 24 | 3.2 |

* Participants could select more than one response

^#^ only the female population is considered when determining the proportion

^†^ only the male population is considered when determining the proportion

^ percentages are only for those respondents who currently smoke

**Appendix B**

**Additional information about the Research Team and Stakeholder Reference Group**

The Research Team described in the methods section comprised two academic researchers, four respiratory specialists, one public health specialist and one representative from a peak body. The Stakeholder Reference Group comprised (one academic researcher, one public health specialist, three representatives from a peak body, one general practitioner, one radiologist).

**Additional information about DCE modelling:**

Additional models were estimated using cost as a continuous variable (see Appendix C). Although there were slight differences in the WTP estimates, these models did not differ substantially from those using cost as a categorical variable. The decision to use models with cost as a categorical variable as the final models was based on the coefficients of the cost levels ($50 and $100).

Based on the results from the MIXL model using cost as a categorical variable (Appendix B - Table B2), the decrements of the two cost levels ($50 and $100) were -1.913 and -1.836 respectively. This suggests that participants perceived no significant difference between the $50 and $100 cost levels, indicating the absence of a clear linear relationship between these two cost levels. Hence, the models using cost as a categorical variable were used to calculate WTP estimates.

The uptake rates were estimated using the MNL model with opt out by calculating the choice probabilities of the most and least preferred programs. This involved taking the exponent of the utility for screening and dividing it by the sum of the exponents of the utilities for both screening and no screening.


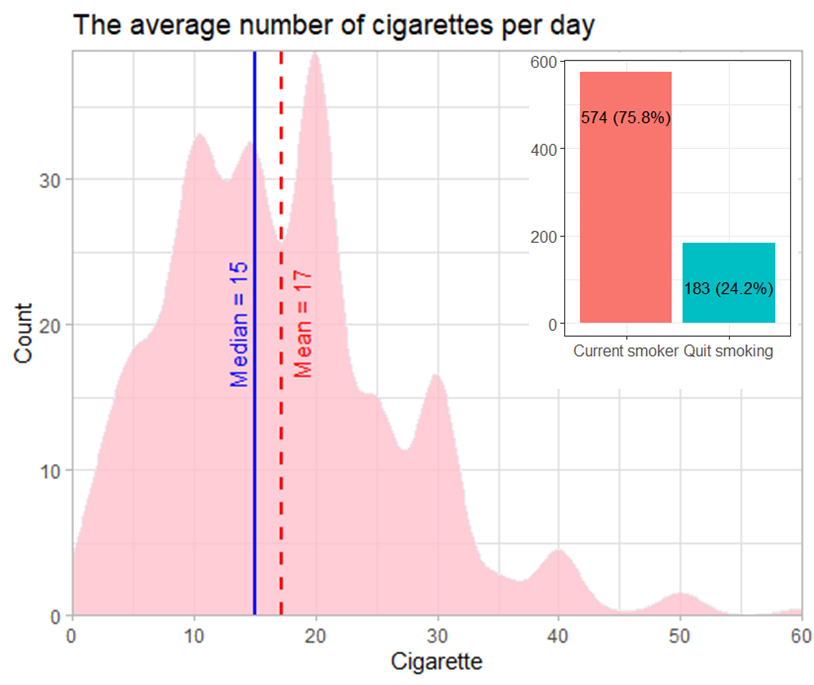


**Figure B1: The average number of cigarettes per day for all participants**


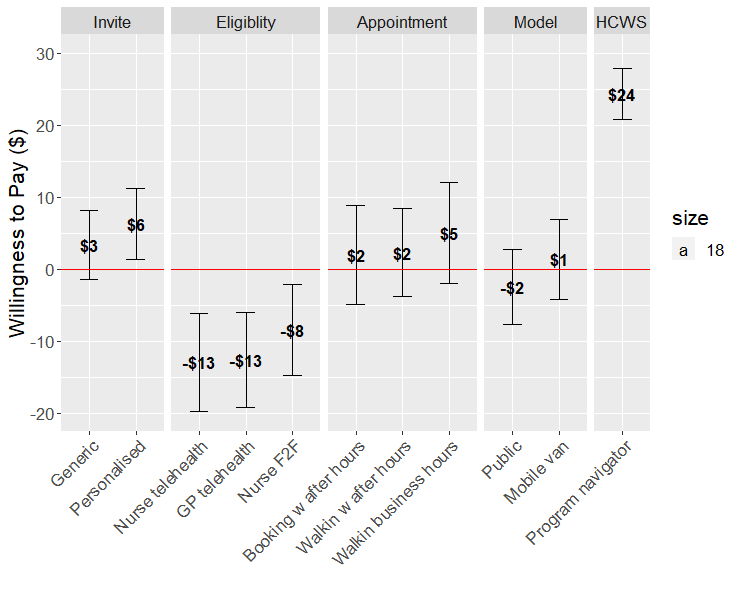


**Figure B2. Willingness to pay estimates based on MNL results**

**Table B1. MNL results with cost coded as categorical**

|  |  | | Coef. | SE | P. |
| --- | --- | --- | --- | --- | --- |
| Invite to screen | | |  |  |  |
|  | Generic Letter or SMS reminder | | 0.056 | 0.040 | 0.167 |
|  | **Personalised invitation (letter or SMS)** | | **0.103** | **0.042** | **0.014** |
| Appointment Booking | | |  |  |  |
|  | Walk in availability, 9am-5pm | | 0.083 | 0.059 | 0.161 |
|  | Walk in availability, with after-hours availability | | 0.038 | 0.051 | 0.460 |
|  | Book phone/online, with after-hours availability | | 0.033 | 0.058 | 0.573 |
| Model of care | | |  |  |  |
|  | Mobile van within local community or township | | 0.023 | 0.047 | 0.629 |
|  | Radiology outpatient at community health service or public hospital | | -0.040 | 0.044 | 0.363 |
| Health care worker support | | |  |  |  |
|  | **A screening program navigator** | | **0.401** | **0.030** | **<0.001** |
| Eligibility Assessment | | |  |  |  |
|  | **Nurse or other health care provider via phone/telehealth** | | **-0.212** | **0.057** | **<0.001** |
|  | **Nurse or other health care provider face to face** | | **-0.139** | **0.053** | **0.009** |
|  | **GP via phone/telehealth** | | **-0.206** | **0.055** | **<0.001** |
| Out-of-pocket Cost of Scan | | |  |  |  |
|  | | **$50** | **-1.647** | **0.141** | **<0.001** |
|  | | **$100** | **-1.627** | **0.044** | **<0.001** |
| Number of observations | | |  |  | 7570 |
| AIC | | |  |  | 7797 |
| BIC | | |  |  | 7887 |
| LL | | |  |  | -3885 |

Note. Significant attribute levels were bolded. Coef: coefficient estimate; SE: standard error; AIC: Akaike’s information criteria; BIC: Bayesian information criteria; LL: Log likelihood.


**Table B2. Sample characteristics and smoking status by class in latent class analysis**

|  |  | All (%) | Class 1 (%) | Class 2 (%) |
| --- | --- | --- | --- | --- |
| Age | 50-54 years | 30.0 | 27.3 | 35.7 |
|  | 55-59 years | 25.4 | 27.1 | 21.7 |
|  | 60-64 years | 24.8 | 25.0 | 24.6 |
|  | 65-70 years | 19.8 | 20.7 | 18.0 |
| Sex | Male | 48.6 | 51.1 | 43.4 |
|  | Female | 51.4 | 48.9 | 56.6 |
| Ethnicity | Indigenous | 3.8 | 3.7 | 4.1 |
|  | Non-Indigenous | 96.2 | 96.3 | 95.9 |
| Country of birth | Australia | 81.4 | 81.1 | 82.0 |
|  | Overseas | 18.6 | 18.9 | 18.0 |
| Education | Completed Year 10 or below | 24.8 | 26.3 | 21.7 |
|  | Completed high school or overseas equivalent | 18.8 | 18.5 | 19.3 |
|  | Other training in addition to high school completion | 31.8 | 32.2 | 31.1 |
|  | Undergraduate degree or higher | 23.6 | 22.2 | 26.6 |
| Region | Metro | 51.1 | 50.3 | 52.9 |
|  | Regional | 48.9 | 49.7 | 47.1 |
| Smoking Status |  |  |  |  |
| Smoking | I currently smoke daily | 75.8 | 76 | 75.4 |
|  | I have quit smoking within the last 10 years | 24.2 | 24 | 24.6 |
| Median daily consumption (per cigarette) excluding people who had quit smoking | | 15 | 15 | 15 |
| Participation in other screening programs | | 62.5 | 58.9 | 70.1 |

Note. Significant attribute levels were bolded. Coef: coefficient estimate; SE: standard error; AIC: Akaike’s information criteria; BIC: Bayesian information criteria; LL: Log likelihood. Class 1: low cost and navigator group; Class 2: tailored navigation group’

**Appendix C**

**Table C1. MNL results with cost coded as continuous**

|  |  | Coef. | SE | P. |
| --- | --- | --- | --- | --- |
| Invite to screen | |  |  |  |
|  | Generic Letter or SMS reminder | 0.058 | 0.040 | 0.147 |
|  | Personalised invitation (letter or SMS) | **0.150** | **0.041** | **<0.001** |
| Appointment Booking | |  |  |  |
|  | Walk in availability, 9am-5pm | -0.057 | 0.054 | 0.293 |
|  | Walk in availability, with after-hours availability | 0.054 | 0.052 | 0.293 |
|  | Book phone/online, with after-hours availability | **-0.128** | **0.051** | **0.011** |
| Model of care | |  |  |  |
|  | Mobile van within local community or township | **-0.089** | **0.043** | **0.038** |
|  | Radiology outpatient at community health service or public hospital | -0.006 | 0.044 | 0.892 |
| Health care worker support | |  |  |  |
|  | A screening program navigator | **0.419** | **0.030** | **<0.001** |
| Eligibility Assessment | |  |  |  |
|  | Nurse or other health care provider via phone/telehealth | -0.057 | 0.050 | 0.255 |
|  | Nurse or other health care provider face to face | **-0.106** | **0.053** | **0.048** |
|  | GP via phone/telehealth | -0.042 | 0.048 | 0.378 |
| Out-of-pocket Cost of Scan | | **-0.017** | **<0.001** | **<0.001** |
| Number of observations | |  |  | 7570 |
| AIC | |  |  | 7827 |
| BIC | |  |  | 7911 |
| LL | |  |  | -3902 |

Note. Significant attribute levels were bolded. Coef: coefficient estimate; SE: standard error; AIC: Akaike’s information criteria; BIC: Bayesian information criteria; LL: Log likelihood.

**Table C2. MIXL results with cost coded as continuous**

|  |  | | Coef. | SE | P. |
| --- | --- | --- | --- | --- | --- |
| Invite to screen | | |  |  |  |
|  | Generic Letter or SMS reminder | | 0.046 | 0.045 | 0.305 |
|  | **Personalised invitation (letter or SMS)** | | **0.183** | **0.046** | **<0.001** |
| Appointment Booking | | |  |  |  |
|  | Walk in availability, 9am-5pm | | -0.047 | 0.057 | 0.405 |
|  | Walk in availability, with after-hours availability | | 0.046 | 0.054 | 0.402 |
|  | **Book phone/online, with after-hours availability** | | **-0.130** | **0.055** | **0.018** |
| Model of care | | |  |  |  |
|  | **Mobile van within local community or township** | | **-0.116** | **0.050** | **0.021** |
|  | Radiology outpatient at community health service or public hospital | | -0.031 | 0.048 | 0.509 |
| Health care worker support | | |  |  |  |
|  | **A screening program navigator** | | **0.468** | **0.041** | **<0.001** |
| Eligibility Assessment | | |  |  |  |
|  | Nurse or other health care provider via phone/telehealth | | -0.082 | 0.056 | 0.144 |
|  | **Nurse or other health care provider face to face** | | **-0.157** | **0.059** | **0.008** |
|  | GP via phone/telehealth | | -0.073 | 0.052 | 0.161 |
| Out-of-pocket Cost of Scan | | | **-0.018** | **0.001** | **<0.001** |
| **Standard Deviation** | | |  |  |  |
| Invite to screen | | |  |  |  |
|  | | Generic Letter or SMS reminder | 0.000 | 0.083 | 0.997 |
|  | | Personalised invitation (letter or SMS) | 0.224 | 0.149 | 0.133 |
| Appointment Booking | | |  |  |  |
|  | | Walk in availability, 9am-5pm | 0.004 | 0.090 | 0.960 |
|  | | Walk in availability, with after-hours availability | 0.028 | 1.023 | 0.979 |
|  | | Book phone/online, with after-hours availability | 0.005 | 0.087 | 0.954 |
| Model of care | | |  |  |  |
|  | | **Mobile van within local community or township** | **0.531** | **0.067** | **<0.001** |
|  | | Radiology outpatient at community health service or public hospital | 0.012 | 0.305 | 0.970 |
| Health care worker support | | |  |  |  |
|  | | **A screening program navigator** | **0.688** | **0.051** | **<0.001** |
| Eligibility Assessment | | |  |  |  |
|  | | Nurse or other health care provider via phone/telehealth | 0.045 | 0.262 | 0.865 |
|  | | Nurse or other health care provider face to face | 0.008 | 0.164 | 0.962 |
|  | | GP via phone/telehealth | 0.000 | 0.132 | 0.999 |
| Number of observations | | |  |  | 7570 |
| AIC | | |  |  | 7730 |
| BIC | | |  |  | 7890 |
| LL | | |  |  | -3842 |

Note. Significant attribute levels were bolded. Coef: coefficient estimate; SE: standard error; AIC: Akaike’s information criteria; BIC: Bayesian information criteria; LL: Log likelihood.

**Table C3. MNL results including the opt out option with cost coded as categorical**

|  |  | | Coef. | SE | P. |
| --- | --- | --- | --- | --- | --- |
| Invite to screen | | |  |  |  |
|  | Generic Letter or SMS reminder | | 0.043 | 0.041 | 0.294 |
|  | **Personalised invitation (letter or SMS)** | | **0.126** | **0.043** | **0.003** |
| Appointment Booking | | |  |  |  |
|  | Walk in availability, 9am-5pm | | 0.028 | 0.053 | 0.593 |
|  | Walk in availability, with after-hours availability | | 0.057 | 0.051 | 0.265 |
|  | Book phone/online, with after-hours availability | | -0.028 | 0.053 | 0.591 |
| Model of care | | |  |  |  |
|  | Mobile van within local community or township | | -0.028 | 0.046 | 0.540 |
|  | Radiology outpatient at community health service or public hospital | | 0.010 | 0.044 | 0.814 |
| Health care worker support | | |  |  |  |
|  | **A screening program navigator** | | **0.448** | **0.032** | **<0.001** |
| Eligibility Assessment | | |  |  |  |
|  | **Nurse or other health care provider via phone/telehealth** | | **-0.137** | **0.052** | **0.008** |
|  | **Nurse or other health care provider face to face** | | **-0.141** | **0.052** | **0.006** |
|  | **GP via phone/telehealth** | | **-0.136** | **0.048** | **0.005** |
| Out-of-pocket Cost of Scan | | |  |  |  |
|  | | **$50** | **-1.231** | **0.056** | **<0.001** |
|  | | **$100** | **-1.716** | **0.046** | **<0.001** |
| **No screening (opt out)** | | | **-1.292** | **0.068** | **<0.001** |
| Number of observations | | |  |  | 7570 |
| AIC | | |  |  | 13440 |
| BIC | | |  |  | 13537 |
| LL | | |  |  | -6706 |

Note. Significant attribute levels were bolded. Coef: coefficient estimate; SE: standard error; AIC: Akaike’s information criteria; BIC: Bayesian information criteria; LL: Log likelihood.


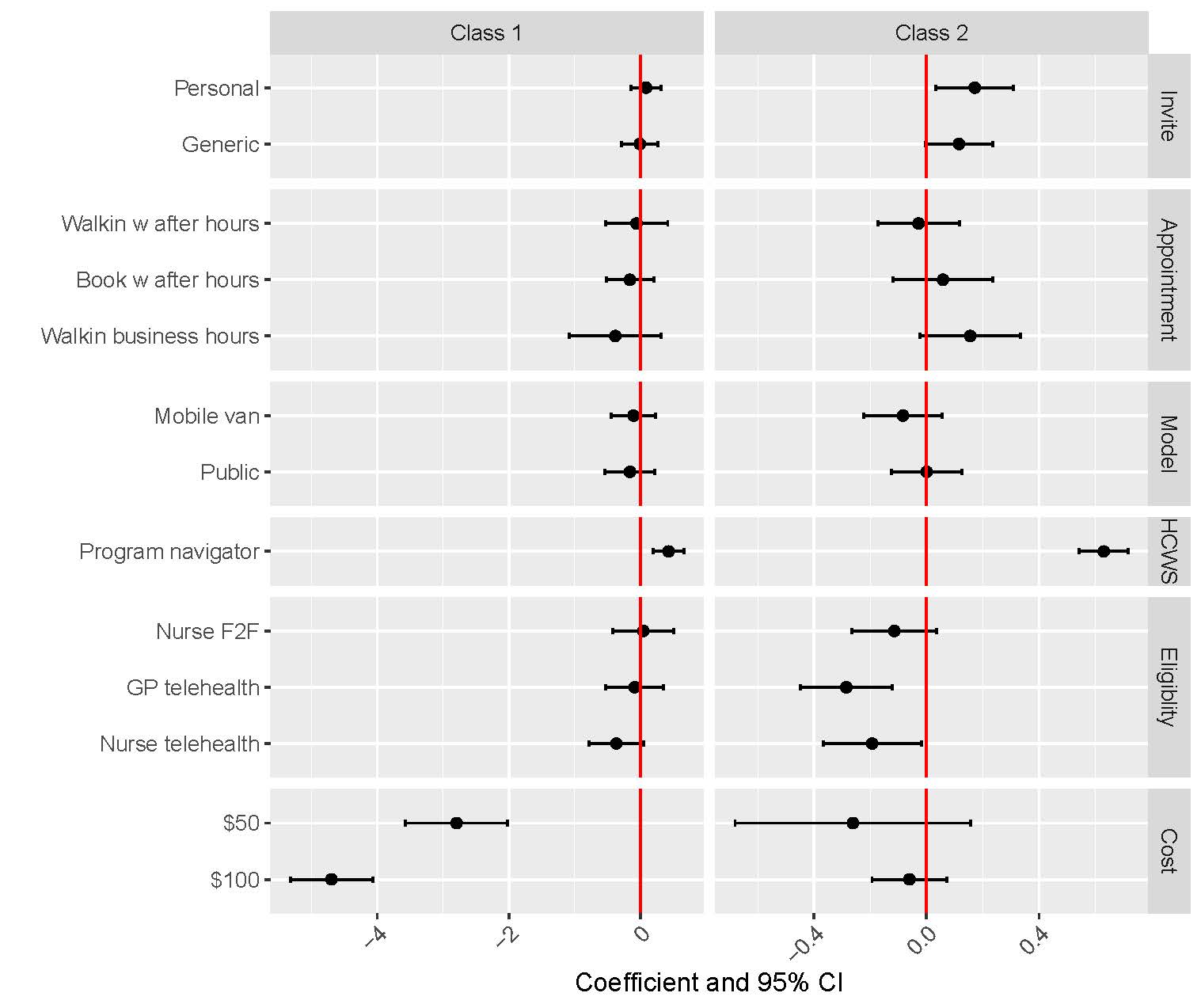
**Figure B3: Latent class analysis**

Note: Class 1 is the 'low cost and navigator group’ and Class 2 is the ‘tailored navigation group’.

The error bars represent the 95% confidence intervals for the estimated coefficients. Values with confidence intervals that do not include 0 are statistically significant compared to the baseline. Values above 0 indicate a positive deviation (preference), while values below 0 indicate a negative deviation (dislike).

Appendix D

**Table D1. MNL with age as an interaction term**

|  |  |  |  |  | Interaction: Age >=60 | | |
| --- | --- | --- | --- | --- | --- | --- | --- |
|  |  | Coef. | SE | P. | Coef. | SE | P. |
| Invite to screen | |  |  |  |  |  |  |
|  | Generic Letter or SMS reminder | **0.143** | **0.055** | **0.009** | **-0.194** | **0.081** | **0.016** |
|  | Personalised invitation (letter or SMS) | **0.206** | **0.057** | **<0.001** | **-0.229** | **0.085** | **0.007** |
| Appointment Booking | |  |  |  |  |  |  |
|  | Walk in availability, 9am-5pm | 0.123 | 0.080 | 0.125 | -0.085 | 0.119 | 0.475 |
|  | Walk in availability, with after-hours availability | -0.003 | 0.069 | 0.969 | 0.109 | 0.104 | 0.295 |
|  | Book phone/online, with after-hours availability | 0.038 | 0.080 | 0.634 | -0.008 | 0.116 | 0.947 |
| Model of care | |  |  |  |  |  |  |
|  | Mobile van within local community or township | 0.041 | 0.064 | 0.520 | -0.037 | 0.094 | 0.694 |
|  | Radiology outpatient at community health service or public hospital | -0.075 | 0.059 | 0.205 | 0.088 | 0.089 | 0.326 |
| Health care worker support | |  |  |  |  |  |  |
|  | A screening program navigator | **0.400** | **0.041** | **<0.001** | 0.012 | 0.060 | 0.840 |
| Eligibility Assessment | |  |  |  |  |  |  |
|  | Nurse or other health care provider via phone/telehealth | **-0.247** | **0.078** | **0.002** | 0.069 | 0.115 | 0.550 |
|  | Nurse or other health care provider face to face | **-0.177** | **0.072** | **0.014** | 0.067 | 0.106 | 0.526 |
|  | GP via phone/telehealth | **-0.263** | **0.075** | **<0.001** | 0.140 | 0.112 | 0.213 |
| Out-of-pocket Cost of Scan | |  |  |  |  |  |  |
|  | $50 | **-1.816** | **0.190** | **<0.001** | 0.401 | 0.284 | 0.158 |
|  | $100 | **-1.635** | **0.058** | **<0.001** | 0.011 | 0.088 | 0.901 |
| Number of observations | |  |  |  |  |  | 7570 |
| AIC | |  |  |  |  |  | 7807 |
| BIC | |  |  |  |  |  | 7988 |
| LL | |  |  |  |  |  | -3878 |

Note. Significant attribute levels were bolded. Coef: coefficient estimate; SE: standard error; AIC: Akaike’s information criteria; BIC: Bayesian information criteria; LL: Log likelihood.

**Table D2. MNL with sex assigned at birth as an interaction term**

|  |  |  |  |  | Interaction: Female | | |
| --- | --- | --- | --- | --- | --- | --- | --- |
|  |  | Coef. | SE | P. | Coef. | SE | P. |
| Invite to screen | |  |  |  |  |  |  |
|  | Generic Letter or SMS reminder | 0.088 | 0.057 | 0.123 | -0.069 | 0.081 | 0.394 |
|  | Personalised invitation (letter or SMS) | 0.098 | 0.060 | 0.100 | 0.015 | 0.084 | 0.861 |
| Appointment Booking | |  |  |  |  |  |  |
|  | Walk in availability, 9am-5pm | -0.008 | 0.086 | 0.925 | 0.176 | 0.118 | 0.138 |
|  | Walk in availability, with after-hours availability | -0.023 | 0.073 | 0.750 | 0.114 | 0.103 | 0.267 |
|  | Book phone/online, with after-hours availability | -0.048 | 0.084 | 0.570 | 0.146 | 0.116 | 0.210 |
| Model of care | |  |  |  |  |  |  |
|  | Mobile van within local community or township | 0.071 | 0.066 | 0.288 | -0.093 | 0.094 | 0.324 |
|  | Radiology outpatient at community health service or public hospital | 0.003 | 0.063 | 0.966 | -0.080 | 0.088 | 0.363 |
| Health care worker support | |  |  |  |  |  |  |
|  | A screening program navigator | **0.410** | **0.042** | **<0.001** | -0.022 | 0.060 | 0.712 |
| Eligibility Assessment | |  |  |  |  |  |  |
|  | Nurse or other health care provider via phone/telehealth | **-0.245** | **0.082** | **0.003** | 0.066 | 0.115 | 0.565 |
|  | Nurse or other health care provider face to face | **-0.167** | **0.075** | **0.026** | 0.053 | 0.106 | 0.619 |
|  | GP via phone/telehealth | **-0.188** | **0.078** | **0.016** | -0.033 | 0.111 | 0.765 |
| Out-of-pocket Cost of Scan | |  |  |  |  |  |  |
|  | $50 | **-1.531** | **0.196** | **<0.001** | -0.231 | 0.282 | 0.412 |
|  | $100 | **-1.559** | **0.061** | **<0.001** | -0.142 | 0.087 | 0.105 |
| Number of observations | |  |  |  |  |  | 7570 |
| AIC | |  |  |  |  |  | 7811 |
| BIC | |  |  |  |  |  | 7991 |
| LL | |  |  |  |  |  | -3879 |

Note. Significant attribute levels were bolded. Coef: coefficient estimate; SE: standard error; AIC: Akaike’s information criteria; BIC: Bayesian information criteria; LL: Log likelihood.

**Table D3. MNL results with remoteness as an interaction term**

|  |  |  |  |  | Interaction: Regional | | |
| --- | --- | --- | --- | --- | --- | --- | --- |
|  |  | Coef. | SE | P. | Coef. | SE | P. |
| Invite to screen | |  |  |  |  |  |  |
|  | Generic Letter or SMS reminder | 0.032 | 0.057 | 0.571 | 0.046 | 0.083 | 0.581 |
|  | Personalised invitation (letter or SMS) | **0.175** | **0.060** | **0.004** | -0.142 | 0.087 | 0.105 |
| Appointment Booking | |  |  |  |  |  |  |
|  | Walk in availability, 9am-5pm | 0.018 | 0.080 | 0.822 | 0.166 | 0.124 | 0.181 |
|  | Walk in availability, with after-hours availability | 0.042 | 0.071 | 0.552 | 0.050 | 0.107 | 0.641 |
|  | Book phone/online, with after-hours availability | -0.079 | 0.075 | 0.293 | **0.251** | **0.122** | **0.039** |
| Model of care | |  |  |  |  |  |  |
|  | Mobile van within local community or township | -0.009 | 0.063 | 0.892 | 0.045 | 0.097 | 0.642 |
|  | Radiology outpatient at community health service or public hospital | -0.011 | 0.063 | 0.856 | -0.076 | 0.091 | 0.400 |
| Health care worker support | |  |  |  |  |  |  |
|  | A screening program navigator | **0.365** | **0.042** | **<0.001** | 0.071 | 0.062 | 0.248 |
| Eligibility Assessment | |  |  |  |  |  |  |
|  | Nurse or other health care provider via phone/telehealth | **-0.169** | **0.078** | **0.030** | -0.091 | 0.120 | 0.446 |
|  | Nurse or other health care provider face to face | **-0.146** | **0.074** | **0.048** | 0.047 | 0.109 | 0.664 |
|  | GP via phone/telehealth | **-0.182** | **0.082** | **0.026** | -0.069 | 0.116 | 0.549 |
| Out-of-pocket Cost of Scan | |  |  |  |  |  |  |
|  | $50 | **-1.325** | **0.207** | **<0.001** | **-0.631** | **0.291** | **0.030** |
|  | $100 | **-1.603** | **0.061** | **<0.001** | -0.063 | 0.089 | 0.483 |
| Number of observations | |  |  |  |  |  | 7570 |
| AIC | |  |  |  |  |  | 7810 |
| BIC | |  |  |  |  |  | 7990 |
| LL | |  |  |  |  |  | -3879 |

Note. Significant attribute levels were bolded. Coef: coefficient estimate; SE: standard error; AIC: Akaike’s information criteria; BIC: Bayesian information criteria; LL: Log likelihood.
